# Validating nonverbal cues for assessing physician empathy in telemedicine: a Delphi study

**DOI:** 10.1080/10872981.2025.2497328

**Published:** 2025-05-08

**Authors:** Annisa Ristya Rahmanti, Hsuan-Chia Yang, Chih-Wei Huang, Ching-Tzu Huang, Lutfan Lazuardi, Che-Wei Lin, Yu-Chuan Jack Li

**Affiliations:** a Department of Health Policy and Management, Faculty of Medicine, Public Health and Nursing, Universitas Gadjah Mada, Yogyakarta, Indonesia; b Department of Computer Science, Faculty of Science and Technology, Middlesex University, London, UK; c International Center for Health Information and Technology, College of Medical Science and Technology, Taipei Medical University, Taipei, Taiwan; d Graduate Institute of Biomedical Informatics, College of Medical Science and Technology, Taipei Medical University, Taipei, Taiwan; e Clinical Big Data Research Center, Taipei Medical University Hospital, Taipei, Taiwan; f Research Center of Big Data and Meta-analysis, Wanfang Hospital, Taipei Medical University, Taipei, Taiwan; g Center for Education in Medical Simulation, Taipei Medical University, Taipei, Taiwan; h Department of Education and Humanities in Medicine, School of Medicine, College of Medicine, Taipei Medical University, Taipei, Taiwan; i Department of Medical Education, Taipei Medical University Shuang Ho Hospital, New Taipei, Taiwan; j Department of Dermatology, Taipei Municipal Wanfang Hospital, Taipei Medical University, Taipei, Taiwan; k The International Medical Informatics Association (IMIA), Geneva, Switzerland

**Keywords:** Nonverbal communication, telemedicine, doctor–patient interaction, Delphi study, empathy

## Abstract

Nonverbal communication is essential in physician–patient interaction, especially in telemedicine where verbal cues may be limited. This study aimed to identify and validate key nonverbal cues for assessing physician empathy in telemedicine consultations through a Delphi method. A three-round Delphi study was conducted from June to November 2022, involving various experts, including academics, healthcare professionals, AI/telemedicine researchers, industry professionals, and patients. Experts evaluated the importance, validity, and reliability of potential nonverbal cues. Consensus was determined based on median responses and expert scoring percentages, with statistical agreement and stability assessed using Kendall’s coefficient of concordance (Kendall’s W) and Wilcoxon signed-rank test. Analyses were conducted using SPSS, version 23.0 with significance set at *p* < 0.05. Of the 72 experts invited, 37 (51%) agreed to participate, with 35 completing the first round (95% completion rate). Eight significant nonverbal cues were identified in the first round, though one did not reach consensus. The second round obtained an 89% response rate (31/35), with three new cues introduced; one did not reach consensus. Round 3 achieved a 94% response rate (29/31), finalizing nine key cues: facial expression, eye contact, tone of voice, smiling, head nodding, body posture, hand gesture, distance, and environmental cues. Among these, facial expression, eye contact, and tone of voice were identified as the most crucial. Inter-expert agreement was statistically significant across all items with strong agreement on the importance (W = 0.739, *p* < 0.001), good agreement on their validity (W = 0.689, *p* < 0.001), and moderate agreement on their reliability (W = 0.452, *p* < 0.001). This study highlights the importance of specific nonverbal cues in telemedicine, particularly facial expression, eye contact, and tone of voice. It provides a validated foundation for developing tools to enhance physician–patient interactions and potentially improve health outcomes in telemedicine.

## Introduction

The widespread adoption of telemedicine due to the COVID-19 pandemic has highlighted the importance of effective doctor-patient communication and the operational challenges it presents for healthcare management [1,2]. Initially developed to bridge geographical barriers in healthcare access, telemedicine has evolved beyond basic remote consultations to encompass telesurgery, tele-diagnosis, robot-assisted procedures, and patient monitoring across various medical fields [3]. Today, telemedicine is widely used in both emergency and non-emergency care, including chronic disease management, mental health, dermatology, antenatal care, and post-operative follow-ups [4–9]. Advances in digital health technologies, artificial intelligence, and wearable devices have further expanded its role, enabling continuous and remote healthcare delivery [4].

While telemedicine enhances healthcare accessibility, reduces travel burdens, and allows for timely medical interventions, it also presents challenges compared to traditional in-person consultations [4]. The lack of physical examination and limited nonverbal cues may hinder accurate diagnosis and physician–patient relationship [10]. Additionally, technological barriers, including unequal access to digital tools and concerns over data security, can impact its effectiveness, particularly in rural and underserved populations [3]. Other limitations include regulatory and legal challenges, which vary across jurisdictions. Barriers related to patient acceptance and adoption may also impact the effectiveness of telemedicine services and reduce overall patient engagement with telehealth services [4].

As telemedicine has enabled continued access to care, it has also transformed traditional doctor–patient interactions [9], requiring new approaches to maintain empathy and patient engagement in remote settings. From a healthcare management perspective, ensuring the quality of telemedicine services is crucial for maintaining patient satisfaction and adherence to treatment plans, both of which are linked to clinical outcomes [11,12]. However, telemedicine introduces complexities, as healthcare providers must rely on limited verbal and nonverbal cues to convey empathy, an essential component of quality care [10].

In this evolving context, the ability to assess and train physicians on nonverbal communication becomes a key quality assurance metric for healthcare organizations [13]. Telemedicine programs that fail to account for the nuances of nonverbal communication may inadvertently reduce the perceived empathy of physicians, leading to lower patient satisfaction and, ultimately, poorer health outcomes [14]. Management strategies that integrate nonverbal communication assessments can help mitigate these risks by providing telemedicine staff with the tools necessary to deliver high-quality, patient-centered care [9,15].

Empathy, a cornerstone of effective physician-patient communication, is defined as the ability to experience and understand a patient’s emotion and concerns [16]. In telemedicine, where physical presence is limited, nonverbal cues – such as facial expressions, eye contact, and tone of voice – play a vital role in maintaining empathetic connections [17]. Healthcare managers need to ensure that these nonverbal cues are appropriately integrated into telemedicine training programs and performance evaluations, fostering a culture of empathetic care across digital consultations [9,10,18].

This study aims to address this need by identifying and validating key nonverbal cues essential for assessing physician empathy in telemedicine through a Delphi study. By leveraging the expert consensus achieved through this method, we equip healthcare managers with actionable insights that can be directly applied to enhancing telemedicine service delivery, quality control, and staff training. Furthermore, the findings offer guidance for medical education by informing the development of training programs that emphasize nonverbal communication skills, crucial for future physicians working in telemedicine and other digital healthcare environments. This aligns with the growing need to prepare healthcare professionals for evolving digital practices in patient care.

## Methods

We conducted a 3-round Delphi study from June 13 to 8 November 2022 to reach a consensus on the ideal core component of nonverbal communication assessment for doctor–patient interaction during telemedicine services. A Delphi study is an effective method for obtaining a consensus among experts in fields where direct evidence may be limited [19]. This technique has been widely adopted in many healthcare and social science studies, especially in situations where there is insufficient information and needs group communication between experts to attain consensus, decision making, problem-solving, and prioritization [20].

### Panel selection

To ensure methodological rigor and transparency, we followed the ACCORD guidelines for panelist recruitment, eligibility criteria, and panel composition [21]. Delphi studies usually consist of 15–30 participants with the same discipline or shared professional background [20]. However, some suggest that 8–12 participants may be sufficient to minimize error and maximize reliability in achieving consensus [22,23]. Given the interdisciplinary nature of our study and the need for global representation, we identified 72 participants through multiple professional networks:

- Faculty/Academic experts, healthcare professionals, and Telemedicine experts were identified from the World Top 2% Scientists in Medical Education and Medical Informatics 2022 Elsevier List, AMEE Technology Enhanced Learning (TEL) Committee, and International Partnership for Health Informatics Education (IPHIE) members.
- Industry experts and patients were recruited via Taipei Medical University (Office of Global Engagement and International Center for Health Information Technology) Networks.

To ensure panel diversity and expertise, we used purposive sampling to select participants based on predefined eligibility criteria. Only those meeting the following inclusion criteria were invited to participate:

- Faculty/Academic: with experience at least 5 years of experience in medical informatics or medical education
- Physician/Nurse/Midwife/Psychologist/Pharmacist/Dentist/Dietitian: with clinical experience at least 3 years and has provided telemedicine video consultation services
- Researcher: actively teaching or conducting research in Artificial Intelligence (emotion recognition) or telemedicine with at least 5 years of experience
- Industry/company: working in Artificial Intelligence (emotion recognition) or telemedicine/telehealth services
- Patient: having used telemedicine video consultation at least 1 session
- An understanding of the purpose of the study and willingness to participate
- Participants should be 20 years or above.

Panelists remained anonymous and were unaware of other panelists’ identities and feedback to reduce potential biases. Only the research team administrator had access to the panelist list to maintain confidentiality. Additionally, peer nominations were not allowed, and no research team members participated as Delphi panelists. No financial incentives were provided, ensuring impartiality in participation.

### Instrument development

**Figure 1.**
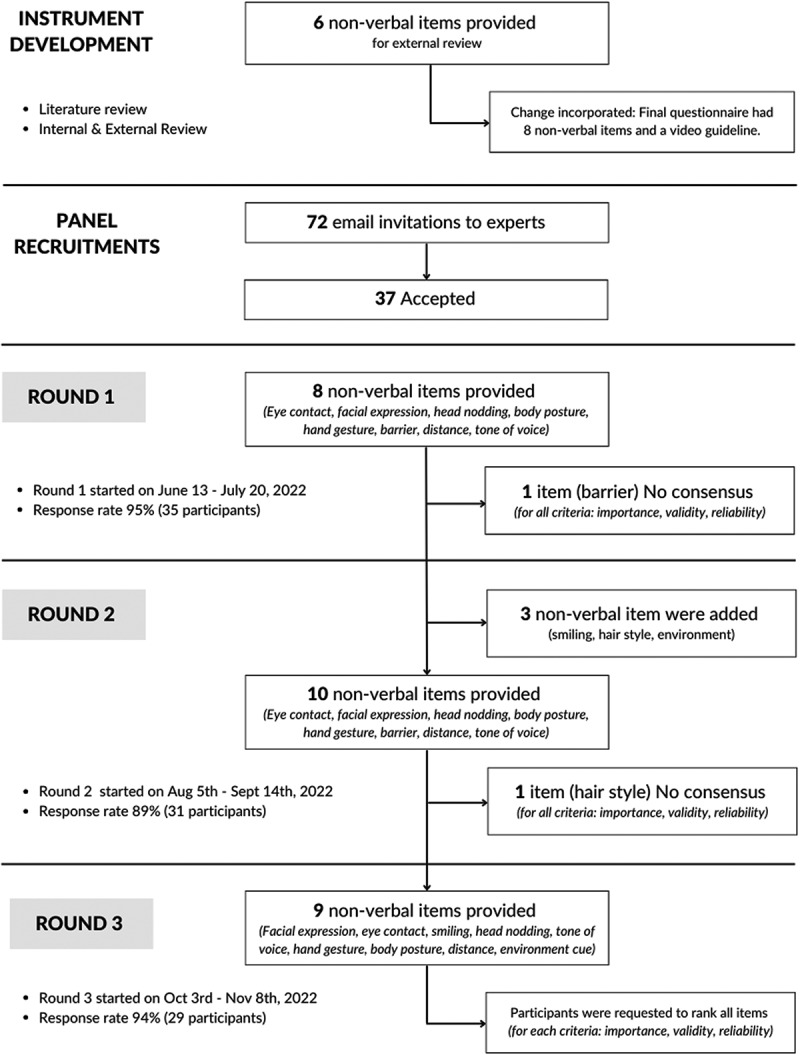
DELPHI study workflow.

The Delphi questionnaire was developed through a literature review and expert consultation, followed by iterative refinement and internal and external review to ensure clarity and usability (Figure 1). Based on the literature review and initial review conducted by the research team, we initially proposed six nonverbal cue items for accessing physician empathy such as ‘eye contact’, ‘facial expression’, ‘body posture’, ‘barrier’, ‘distance’, and ‘tone of voice.’

To further refine the questionnaire, a simulation trial was conducted with 15 external reviewers (physicians and researchers familiar with AI and telemedicine) who were not part of the expert panel. This trial evaluated the completion time, clarity of item description, and suggestion for additional nonverbal cues. Following the conclusion of the trial, the final questionnaire has been revised to incorporate eight nonverbal cues, which now include ‘head nodding’ and ‘hand gesture.’ Each of these nonverbal cues was also supplemented with a video demonstration to improve clarity and consistency. Questionnaire rounds 1 and 2 were administered via Google Forms^TM^ (Alphabet, Mountain View, CA, USA), while round 3 used Qualtrics^TM^ (Qualtrics, Provo, UT, USA) to facilitate a user-friendly ranking statement.

### Study workflow

This study utilized a Delphi method and was conducted in English over three rounds of online surveys (Figure 1).

#### First round

Participants were invited via email to complete a web-based questionnaire, rating their agreement towards eight nonverbal cue items on their importance, validity, and reliability on a scale of 1 (lowest) to 5 (highest). Further, participants were asked to justify their agreement rating and suggest new items and provided demographic data and potential conflicts of interest. After collecting response for 2–3 weeks, the feedback was processed to refine the nonverbal cues list for the second round.

#### Second round

Participants received a new questionnaire link featuring the refined list and a summary of the first round’s results. They were asked to reassess the cues using the same Likert scale and provide justification for their ratings. Based on predetermined consensus thresholds, we selected cues for the final round and included feedback for any items that did not reach consensus.

#### Third round

We provided a new questionnaire link containing the final list of nonverbal cues along with a detailed internal report of the second Delphi round and asked the participants to rank each provided cue based on its priority.

The response rates across three rounds of a Delphi study were 95% in the first round 1 with 35 of the 37 invitees participating, 89% in the second round (31 of 35 invitees), and 94% in the third round (29 of 31 invitees).

### Statistical analysis

We used descriptive statistics to describe participants’ demographic characteristics and their responses across all three rounds.

Rating statement criteria for round 1 and round 2:

- **High consensus**: if the median response ≥3, with at least 80% of the experts scoring the item at 3 or above,
- **Low consensus**: if the median response ≥3, but only 70–79% of the experts scored the items at 3 or above, and
- **No consensus**: if the median response <3 and <70% of the experts scored the item at 3 or above.

For ranking statements in round 3, we calculated the mean rank for each nonverbal item based on participant responses, where a lower mean rank indicates a higher priority. We also used Kendall’s coefficient of concordance (Kendall’s W), statistical significance set at *p* < 0.05 to evaluate the inter-expert agreement across all rating statements [24]. Strength of inter-expert agreement (W) measured using

- less than 0.3 weak agreement
- 0.3 to 0.5 moderate agreement
- 0.5 to 0.7 good agreement
- 0.7 to 0.9 strong agreement

Wilcoxon signed rank test was also performed to analyze the stability in the response of experts between round 1 and round 2. Stability of consensus was considered reached if *p* > 0.05. All analysis was conducted using Statistical package for social science (SPSS, version 23.0).

## Results

### Participants

Table 1 showed the demographic characteristics of 35 panels enrolled onto the study. The participants were predominantly academic faculty (31%, *n* = 11), healthcare professionals (19%, *n* = 9), and patient representatives (29%, *n* = 10). In terms of gender distribution was fairly balanced (54% male, *n* = 19 vs. 46% female, *n* = 16), with the most represented age group being 45–54 years (32%, *n* = 11). In the context of telemedicine video consultation familiarity, 43% attending more than three sessions (*n* = 15).

**Table 1.** Baseline characteristics of participants.

Characteristics	Value (n, %)
Panelist classification ● Faculty/Academic in medical informatics/medical education ● Healthcare Professionals ● Researcher in AI or telemedicine/telehealth ● Industry/company ● Patient	11 (31%) 9 (26%) 3 (9%) 2 (6%) 10 (29%)
Gender ● Female ● Male	16 (46%) 19 (54%)
Age group ● 18 to 24 years old ● 25 to 34 years old ● 35 to 44 years old ● 45 to 54 years old ● 55 to 64 years old ● 65 years old or older	2 (6%) 9 (26%) 9 (26%) 11 (32%) 2 (6%) 2 (6%)
Country of residence ● Australia ● Canada ● Indonesia ● Malaysia ● Netherlands ● New Zealand ● Oman ● Portugal ● South Korea ● Taiwan ● UK ● USA ● Vietnam	1 (3%) 1 (3%) 7 (20%) 1 (3%) 1 (3%) 1 (3%) 1 (3%) 1 (3%) 1 (3%) 10 (29%) 1 (3%) 8 (23%) 1 (3%)
Highest academic level ● High School ● Bachelor ● Master ● PhD ● Professional: MD, specialist	1 (3%) 1 (3%) 17 (51%) 11 (34%) 3 (9%)
Occupation (*Participants may select more than 1 option) ● Academic faculty ● Government officials ● Healthcare ProfessionalsIndustry/company ● Researcher ● Student	23 (66%) 3 (9%) 19 (54%) 3 (9%) 13 (37%) 2 (6%)
Number of telemedicine video consultation attended ● 1–3 ● >3 ● None	14 (40%) 15 (43%) 6 (17%)

### Round 1

In the first round, high consensus was achieved on six nonverbal cues – ‘eye contact’, ‘facial expression’, ‘head nodding’, ‘body posture’, ‘hand gesture’, and ‘tone of voice’ – regarding their importance, validity, and reliability. Meanwhile, ‘distance’ achieved high consensus on its importance, but showed varied opinions on validity and reliability. The ‘barrier’ did not reach consensus and was removed for the second round. The inter-expert agreement across all items was statistically significant but weak for the three criteria: importance (W = 0.254, *p* < 0.001), validity (W = 0.194, *p* < 0.001), and reliability (W = 0.140, *p* < 0.001).

### Round 2

In response to expert feedback, two new items were introduced: ‘appearance (hairstyle)’ and ‘environmental cues’ to further explore nonverbal communication’s impact on patient perceptions of physician professionalism and empathy in telemedicine. Additionally, ‘smiling’ was separated from ‘facial expression’ due to the diverse range of emotions smile can convey. Thus, 10 items were evaluated in this round (Supplementary Table S2). Consensus on the importance, validity, and reliability was achieved for several cues. ‘Facial expression’ and ‘eye contact’ consistently showed high agreement across all criteria. ‘Smiling’, ‘tone of voice’, ‘head nodding”, ‘body posture’, and ‘environment’ also reached high consensus. Although ‘hand gesture’ received high consensus on its importance and reliability, its validity received slightly lower ratings. “Distance’ was highly regarded for its importance but had varied opinions regarding its validity and reliability. The ‘hairstyle’ item failing to reach consensus and was therefore removed for the third round.

Kendall’s Coefficient of Concordance showed a statistically significant but weak inter-expert agreement across all the items in terms of importance (W = 0.231, *p* < 0.001), validity (W = 0.247, *p* < 0.001), and reliability (W = 0.195, *p* < 0.001). This finding reinforces that while there is agreement on certain items, variability persists in expert opinions. The stability of responses between the first and second rounds was confirmed by the Wilcoxon signed rank test, which showed no significant differences in expert opinions (*p* > 0.05), suggesting consistent and reliable perspectives across these rounds.

### Round 3

In the third round, the participants evaluated nine nonverbal cues: ‘facial expression’, ‘eye contact’, ‘tone of voice’, ‘smiling’, ‘head nodding’, “body posture, hand gesture, distance, and environmental cues (Table 2). The experts agreed that ‘facial expression’ and ‘eye contact’ were of the highest importance, and ‘Environment’ of the lowest. ‘eye contact’ was considered the most valid, followed by ‘facial expression’. In terms of reliability, ‘facial expression’ ranked highest, with ‘Environment’ again the lowest.

**Table 2.** Expert rankings of nine nonverbal items.

Items	Mean Rank	Priority Order
**Importance** Facial Expression Eye contact Tone of Voice Smiling Head Nodding Body Posture Hand Gesture Distance Environment	1.62 1.83 3.52 4.24 5.41 6.14 6.55 7.72 7.97	1 2 3 4 5 6 7 8 9
**Validity** Eye contact Facial Expression Tone of Voice Smiling Head Nodding Body Posture Hand Gesture Distance Environment	1.93 2.17 3.34 4.52 4.72 5.93 6.52 7.41 8.45	1 2 3 4 5 6 7 8 9
**Reliability** Facial Expression Eye contact Tone of Voice Smiling Head Nodding Body Posture Hand Gesture Distance Environment	2.24 2.52 4.38 4.66 5.07 5.69 5.79 6.66 8.00	1 2 3 4 5 6 7 8 9

In the third round, the experts showed strong agreement on the importance of the items (W = 0.739, *p* < 0.001), good agreement on their validity (W = 0.689, *p* < 0.001), and moderate agreement on their reliability (W = 0.452, *p* < 0.001). All these agreements were statistically significant, indicating reliable consensus.

## Discussions

Our Delphi study identified nine nonverbal cues crucial for assessing physician empathy in telemedicine, which include facial expression, eye contact, tone of voice, smiling, head nodding, body posture, hand gestures, distance, and environmental cues. Among these, facial expression, eye contact, and tone of voice securing high priority ranks from experts in terms of importance, validity, and reliability.

Our conceptual framework systematically categorizes and ranks nonverbal cues based on their impact on conveying empathy, grounding the study in communication theory and psychological models of empathy [25,26]. This framework clarifies the complex roles nonverbal cues play in facilitating empathetic interactions in a telemedicine context, as supported by Bordage (2009), who emphasizes the importance of conceptual frameworks in medical education for enhancing understanding and guiding research [27].

Facial expressions represent a spectrum of emotions through distinct facial movements, are fundamental in communicating emotional states and responses [28,29], as noted in our results where they secured the top rank in both importance and reliability concerning physician empathy. Their ability to convey genuine emotions makes them invaluable in assessing a physician’s empathetic engagement [14], reflecting consistency across diverse cultures and ensuring dependable results in empathy assessment [14,30].

Eye contact stands out in terms of validity, serves as an accurate indicator of empathetic connection in telemedicine [31]. Maintaining eye contact, even during telemedicine, is perceived as a sign of the physician’s active engagement and sincerity [14,31,32]. This perspective aligns with a cross-cultural experimental study by Helou, S et al., which found that physicians who directed their eye contact at the camera were perceived as more attentive and achieved higher communication ratings from participants [31].

Tone of voice is also critical, reflecting emotional nuances that significantly impacting patient–provider rapport [14]. Its effectiveness is influenced by valence (the positivity or negativity of an emotion), dominance (the level of control or assertiveness), and arousal (the intensity of emotion), all of which play pivotal roles in conveying empathy especially in telemedicine – where visual cues may be limited [33–35]. For instance, a gentle tone with positive valence might reassure an anxious patient, fostering trust and confidence [33]. While a tone characterized by high dominance might be suitable for conveying crucial instructions or clarifications, highlighting the versatility and impact of vocal cues in virtual consultations [33].

Meanwhile, genuine pleasantness is often reflected through smiling, a simple yet powerful cue that can immediately establish rapport and trust between a physician and a patient [36,37]. While facial expressions encompass a broader range of emotions and are more complex in their presentation, a smile specifically denotes positivity, approachability, and openness [30,36,38]. On the other hand, head nodding, especially in telemedicine, is pivotal as it offers a visual affirmation of understanding or agreement in the absence of physical presence. In teleconsultations, where verbal cues might sometimes get lost due to technical glitches or delays, head nodding becomes a consistent and reliable nonverbal affirmation, ensuring the patient feels heard and understood [39].

Body posture and positioning in relation to the camera, combined with the appropriateness of hand gestures, also play crucial roles in patient-physician communication [14]. The way a physician positions themselves – whether leaning forward in an engaged manner or leaning back in a potentially disengaged posture – can convey attentiveness, openness, or detachment [29,40]. In telemedicine, these postural cues are magnified due to the limited visual frame, emphasizing the importance of positioning oneself optimally within the camera’s view [40,41]. Similarly, hand gestures can amplify or complement verbal messages, adding depth and enhancing understanding [40,42]. In a virtual setting, excessive or misplaced hand gestures might divert attention, while well-timed gestures can augment the richness of communication, compensating for the physical distance inherent in telemedicine [43].

Finally, the spatial distance from the camera and the consultation environment significantly influence the efficacy of the interaction. When a physician is positioned too close or too far from the camera, it may make the consultation feel intrusive or distant, respectively. An optimal distance fosters a comfortable virtual space, mirroring a face-to-face conversation [44,45]. Additionally, the background environment sets the tone for the consultation. A clear, organized space conveys professionalism and reduces potential distractions, whereas a chaotic environment can detract from the patient’s confidence in the physician’s competence and focus. In telemedicine, where the environment is limited to what is visible in the frame, ensuring a conducive consultation backdrop is essential for effective communication [44].

### Implications for policy, practice, and research in medical education

Understanding and applying the identified nonverbal cues is crucial for enhancing physician–patient interactions in telemedicine. While some may perceive the importance of cues like facial expression, eye contact, and tone of voice as obvious, our study’s systematic approach validates these cues, providing a robust empirical foundation that reinforces that these findings are actionable and not merely intuitive. By emphasizing such cues, physicians can significantly enhance the perception of empathy, building a stronger rapport with their patients even in the absence of physical presence [14].

Recognizing these nonverbal cues lays the groundwork for advancing real-time analytical tools specific to telemedicine dynamics [46]. These tools will offer real-time feedback to clinicians, pinpointing areas for improvement in their nonverbal communication. The integration of such technology into telemedicine practices is expected to significantly increase patient satisfaction and strengthen physician–patient relationships [6,47,48]. Additionally, the specific ranking of nonverbal cues allows physicians to focus on the most effective communication methods within a telemedicine setting, where some cues might be constrained by technical limitations or health precautions like mask-wearing [29].

Moreover, by systematically identifying and validating a set of crucial nonverbal cues and a framework for conveying empathy, our study equips healthcare managers with actionable insights that can be used to enhance telemedicine services. This includes the integration of these cues into physician empathy assessment tools, ensuring that physicians are well equipped to manage patient interactions effectively in a digital healthcare environment. Implementing these findings can help bridge the gap in medical education, particularly in developing nonverbal communication skills tailored to virtual healthcare settings. As telemedicine continues to evolve, our proposed framework serves as a foundation for enhancing the competence of healthcare professionals in telemedicine settings and aligning with broader organizational goals to improve the quality of patient care.

### Strength and limitation

The primary strength of our study relies on its comprehensive international scope, incorporating insights from experts across 13 countries and four continents. This diverse expertise included academia, healthcare professionals, telemedicine experts, industry stakeholders, and patients, ensuring a comprehensive analysis from multiple perspectives. While our panel lacked representation from psychologists specializing in clinical or counseling aspects and experts in speech and linguistics, it did include medical educators experienced in patient-physician communication. These professionals bring a critical perspective that differs from general conversation analysis, focusing specifically on nuances that directly impact patient care and treatment adherence. Nevertheless, the absence of certain specialties might have resulted in overlooking specific nuances, potentially restricting the complete applicability and generalizability of our framework.

Our study specifically focuses on nonverbal communication in physician–patient interactions, where empathy and engagement are key components of care. While nonverbal cues play a role in professional medical collaborations, such as teleconsultations, multidisciplinary team discussions, and remote mentoring, the nature of these interactions differs significantly in terms of emotional exchange and communication objectives. Future research could conduct a detailed exploration of physician-to-physician nonverbal communication to further enrich telemedicine practices.

## Conclusion

In this Delphi study, we identified nine nonverbal cues pivotal for assessing physician empathy in telemedicine, with facial expression, eye contact, and tone of voice highlighted as particularly significant. This research not only advances our understanding of empathetic communication in telemedicine but also sets the groundwork for improving clinical practice and patient outcomes through better training and technology. By creating a structure framework for nonverbal communication, our findings offer valuable tools for healthcare professionals and support the ongoing evolution of telemedicine protocols, significantly contributing to the improvement of healthcare delivery in a digital age.

## Supplementary Material

Supplementary Files.docx
