# Supplementary material for: Validating nonverbal cues for assessing physician empathy in telemedicine: a Delphi study
Source: Med Educ Online. 2025 May 8;30(1):2497328. doi: 10.1080/10872981.2025.2497328 (PMC12064121; doi:10.1080/10872981.2025.2497328)
Supplement: Supplementary Files.docx [file ZMEO_A_2497328_SM7763.docx]

# **Supplementary Files**

**Supplementary Table 1. Consensus Result from DELPHI Round 1**

| **Items** | **Median  (Min & Max Ratings)** | **% experts who scored  at least 3 Points** | **Consensus** |
| --- | --- | --- | --- |
| **Item 1: Eye contact**  1a. Important 1b. Validity  1c. Reliability | 5 (3-5)  4 (2-5)  4 (2-5) | 35 (100.0)  34 (097.1)  34 (097.1) | High  High  High |
| **Item 2: Facial Expression**  2a. Important 2b. Validity  2c. Reliability | 5 (3-5)  4 (2-5)  4 (2-5) | 35 (100.0)  34 (097.1)  33 (094.3) | High  High  High |
| **Item 3: Head Nodding**  3a. Important 3b. Validity  3c. Reliability | 4 (2-5)  4 (2-5)  4 (1-5) | 34 (97.1)  32 (91.4)  31 (88.6) | High  High  High |
| **Item 4: Body Posture**  4a. Important 4b. Validity  4c. Reliability | 4 (1-5)  4 (1-5)  4 (1-5) | 33 (94.3)  30 (85.7)  28 (80.0) | High  High  High |
| **Item 5: Hand Gesture**  5a. Important 5b. Validity  5c. Reliability | 4 (2-5)  4 (1-5)  4 (2-5) | 31 (88.6)  30 (85.7)  28 (80.0) | High  High  High |
| **Item 6: Barrier**  6a. Important 6b. Validity  6c. Reliability | 4 (1-5)  3 (1-5)  3 (1-5) | 24 (68.6)  23 (65.7)  21 (60.0) | No consensus  No consensus  No consensus |
| **Item 7: Distance**  7a. Important 7b. Validity  7c. Reliability | 4 (1-5)  4 (1-5)  4 (1-5) | 30 (85.7)  26 (74.3)  26 (74.3) | High  Low  Low |
| **Item 8: Tone of Voice**  8a. Important 8b. Validity  8c. Reliability | 5 (2-5)  5 (2-5)  5 (1-5) | 30 (85.7)  29 (82.9)  28 (80.0) | High  High  High |

**Supplementary Table 2. Consensus Result from DELPHI Round 2**

| **Items** | **Median  (Min & Max Ratings)** | **% experts who scored  at least 3 Points** |
| --- | --- | --- |
| **Item 1: Facial Expression**  1a. Important 1b. Validity  1c. Reliability | 5 (1-5)  4 (1-5)  4 (1-5) | **29 (93.5) high**  **29 (93.5) high**  **27 (87.1) high** |
| **Item 2: Eye contact**  2a. Important 2b. Validity  2c. Reliability | 5 (1-5)  4 (1-5)  4 (1-5) | **29 (93.5) high**  **27 (87.1) high**  **27 (87.1) high** |
| **Item 3: Smiling**  3a. Important 3b. Validity  3c. Reliability | 4 (1-5)  4 (1-5)  4 (1-5) | **30 (96.8) high**  **27 (86.7) high**  **28 (90.3) high** |
| **Item 4: Tone of Voice**  4a. Important 4b. Validity  4c. Reliability | 5 (2-5)  4 (1-5)  4 (1-5) | **29 (93.5) high**  **25 (80.6) high**  **25 (80.6) high** |
| **Item 5: Head Nodding**  5a. Important 5b. Validity  5c. Reliability | 4 (2-5)  4 (1-5)  4 (1-5) | **28 (90.3) high**  **27 (87.1) high**  **26 (83.9) high** |
| **Item 6: Hand Gesture**  6a. Important 6b. Validity  6c. Reliability | 4 (1-5)  3 (1-5)  3 (1-5) | **25 (80.6) high**  **23 (74.2) low**h  **25 (80.6) high** |
| **Item 7: Body Posture**  7a. Important 7b. Validity  7c. Reliability | 4 (2-5)  4 (1-5)  4 (2-5) | **28 (90.3) high**  **28 (90.3) high**  **27 (87.1) high** |
| **Item 8: Hair Style**  8a. Important 8b. Validity  8c. Reliability | 3 (1-5)  2 (1-5)  3 (1-5) | 16 (51.6) no consensus  15 (48.4) no consensus  16 (51.6) no consensus |
| **Item 9: Distance**  9a. Important 9b. Validity  9c. Reliability | 4 (2-5)  3 (1-5)  3 (2-5) | **27 (87.1) high**  **22 (71.0) lowh**  **23 (74.2) lowh** |
| **Item 10: Environment**  10a. Important 10b. Validity  10c. Reliability | 4 (1-5)  4 (1-5)  4 (1-5) | **28 (90.3) high**  **27 (87.1) high**  **28 (90.3) high** |
